# Additives in Children’s Nutrition—A Review of Current Events

**DOI:** 10.3390/ijerph192013452

**Published:** 2022-10-18

**Authors:** Marijana Savin, Aleksandra Vrkatić, Danijela Dedić, Tomislav Vlaški, Ivana Vorgučin, Jelena Bjelanović, Marija Jevtic

**Affiliations:** 1 Faculty of Medicine, University of Novi Sad, Hajduk Veljkova 3, 21000 Novi Sad, Serbia; 2 Institute for Child and Youth Health Care of Vojvodina, Hajduk Veljkova 10, 21000 Novi Sad, Serbia; 3 Emergency Service, Community Health Center Šid, Alekse Šantića 1, 22239 Šid, Serbia; 4 Institute of Public Health of Vojvodina, Futoška 121, 21000 Novi Sad, Serbia; 5 Research Center on Environmental Health and Occupational Health, School of Public Health, Université Libre de Bruxelles (ULB), 1050 Bruxelles, Belgium

**Keywords:** additives, children, bisphenols, phthalates, perfluoroalkyl chemicals, perchlorate, pesticides, nitrates, nitrites, synthetic food colors, monosodium glutamate, aspartame

## Abstract

Additives are defined as substances added to food with the aim of preserving and improving safety, freshness, taste, texture, or appearance. While indirect additives can be found in traces in food and come from materials used for packaging, storage, and technological processing of food, direct additives are added to food with a special purpose (canning). The use of additives is justified if it is in accordance with legal regulations and does not pose a health or danger to consumers in the prescribed concentration. However, due to the specificity of the child’s metabolic system, there is a greater risk that the negative effects of the additive will manifest. Considering the importance of the potential negative impact of additives on children’s health and the increased interest in the control and monitoring of additives in food for children, we have reviewed the latest available literature available through PubMed, Scopus, and Google Scholar. Expert data were taken from publicly available documents published from January 2010 to April 2022 by internationally recognized professional organizations. It was found that the most frequently present additives in the food consumed by children are bisphenols, phthalates, perfluoroalkyl chemicals, perchlorates, pesticides, nitrates and nitrites, artificial food colors, monosodium glutamate, and aspartame. Increasing literacy about the presence and potential risk through continuous education of parents and young people as well as active monitoring of newly registered additives and harmonization of existing legal regulations by competent authorities can significantly prevent the unwanted effects of additives on children’s health.

## 1. Introduction

Additives are defined as substances added to food with the aim of preserving and improving safety, freshness, taste, texture, or appearance. They are used in the process of industrial production, in order to preserve the quality, that is, the nutritional value of the food that should reach the consumer. They can be of plant, animal, mineral, or synthetic origin [1]. Additives are divided into direct and indirect additives. Direct additives include substances that are added to food with a specific purpose (e.g., preservation). Indirect additives represent those substances that are found in traces in food and come from materials used for packaging, storage, or technological processing of food [2].

The use of food additives is justified only if it is in accordance with current legal regulations, if it does not pose a danger to the health of consumers in the concentration that is allowed, does not mislead consumers, and serves a well-defined technological function. It is also necessary to for additives to have a benefit for the consumer, as they are used in order to preserve the nutritional value of the food, quality, stability, or organoleptic properties of the food, without changing the nature, composition, and quality, as well as to help in production, transport, and storage of food. Additives must not be used to cover up product defects [3]. Over 10,000 different additives have been approved for use in the food industry. The most used groups of additives in children’s food are food colors, sweeteners, preservatives, and flavor enhancers [4]. Research indicates that substances found in food, as well as materials that come into contact with food as part of packaging or processing equipment, can potentially negatively affect children’s health. Previously published studies indicated that bisphenols, phthalates, perfluoroalkyl chemicals, perchlorate, pesticides, nitrates, nitrites, and synthetic food colors cause special concern regarding children’s health [5,6,7,8], so we have included them in this paper. As there are controversial data for aspartame and monosodium glutamate concerning health safety in children’s nutrition, they are included in this review. Table 1 shows the division of additives mentioned in this review, the method of using additives, the proposed mechanism of action, and the potential health risks associated with these additives.

Given the specificity of the metabolic system and significant changes in the body during development, there is a greater risk of negative effects of additives on the body in children. In the first three years of life, the caloric intake per kilogram of body weight is higher, which means that the intake of additives is also higher per kg/body weight (BW) than in an adult [8].

## 2. Methodology

**Figure 1:**
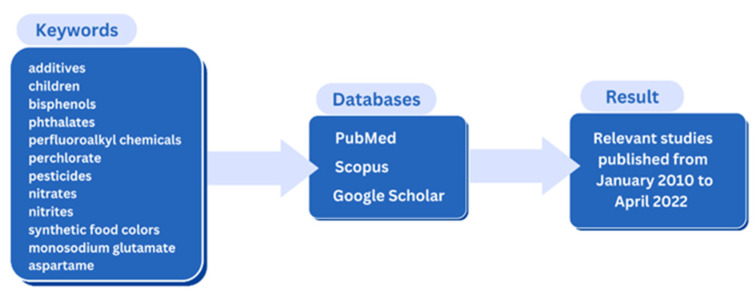
Methodology scheme of this review.

Considering the importance of the potential negative impact of additives on children’s health and the increased interest in the control and monitoring of food additives for children, the most recent available literature was searched. For the purposes of writing the review paper, professional and scientific literature, available through PubMed, Scopus and Google Scholar, were used, with the use of keywords, that is, concepts that are treated in this paper and their combinations: additives, children, bisphenols, phthalates, perfluoroalkyl chemicals, perchlorate, pesticides, nitrates, nitrites, synthetic food colors; monosodium glutamate, and aspartame. Expert data were taken from publicly available documents published from January 2010 to April 2022 by internationally recognized professional organizations. Methodology scheme of this review is shown in Figure 1.

## 3. Indirect Additives in Children’s Nutrition

### 3.1. Bisphenols

Bisphenols are substances used in the production of primary packaging, most often to coat metal cans to prevent corrosion. Bisphenol A (BPA) shows the potential to bind to the estrogen receptor and induce estradiol-like tissue response and is classified as an “endocrine disruptor”. According to the definition by the US Environmental Protection Agency, endocrine disruptors (EDCs) are “exogenous agents that interfere with the synthesis, secretion, transport, metabolism, site of action or elimination of hormones present in the body, which are responsible for homeostasis, reproduction, and growth”. It is considered that EDCs are characterized by a long latent period, the effect of bioaccumulation, transgenerational epigenetic inheritance, in utero and postnatal impact on development, as well as the synergistic effect of several different EDCs [9]. The Centers for Disease Control and Prevention (CDC) found BPA in more than 90 percent of the US population examined, and the highest estimated daily intake of BPA was found in infants and children. Research conducted so far has observed that exposure to BPA is associated with the onset of polycystic ovary syndrome, obesity, and an increase in cardiometabolic risk factors in children [10]. In research in Turkey, which was conducted in the period 2016–2018, it was observed that exposure to BPA can modify neuroendocrine, reproductive, and metabolic regulation favoring the development of polycystic ovary syndrome in adolescent girls [11]. Low dose of BPA promotes reversible epigenetic changes in a key adipogenic gene [12]. In a study of 2838 participants aged 6 to 19 years, higher urinary BPA concentrations were found to be associated with obesity [13]. In a study conducted in Iran at the age of 6–18 years, a statistically significant association was found between BPA exposure and an increase in waist circumference, body mass index, blood pressure, and glycemia in children and adolescents [14].

In 2012, the FDA banned the use of BPA in baby bottles and children’s drinking cups, after several major manufacturers had previously voluntarily removed it. Despite the ban on BPA in baby bottles, infants are still exposed to this substance through maternal exposure during pregnancy or through breast milk. It is recommended that pregnant and lactating women should avoid canned food and carbonated drinks [15]. As the FDA banned the production of bottles with BPA, alternative compounds, bisphenol F and bisphenol S, were synthesized. However, some studies show that newly synthesized compounds can have harmful effects on human health, including children [16].

### 3.2. Phthalates

Phthalates are diphthalic acid esters that are found in lubricants, adhesives, and plasticizers during the packaging manufacturing process. In the research carried out so far, the association of phthalates with the risk of insulin resistance, disorders in the development of the male genital system, premature birth, metabolic disorders, and cardiac disorders have been observed. Metabolism of phthalates produces products that affect the expression of peroxisome proliferator-activated receptors (PPARs), which play a significant role in the metabolism of lipids and carbohydrates, increasing the risk of insulin resistance. Thiazolidine drugs, which act through these receptors, have found therapeutic use in increasing insulin sensitivity [17].

In a study conducted among adolescents aged 12–19 years, in the U.S., the association of di-isononyl phthalate (DINP), di-isodecyl phthalate (DIDP), and di-2-ethyl hexyl phthalate (DEHP) with the onset of insulin resistance was investigated. DINP and DIDP are substances used to replace DEHP and are widely present in processed foods. In the mentioned study, the association of urinary DINP and DEHP, but not DIDP, with the occurrence of increased insulin resistance was established [18]. Monoethylhexyl phthalate is a metabolite of DEHP that is associated with disturbances in lipid and carbohydrate metabolism and contributes to childhood obesity and insulin resistance [19].

Research conducted so far indicates that some phthalates are antiandrogenic and have a negative effect on the development of the male genital system. In a study by Swan et al., it was observed that exposure to DEHP during the first trimester was negatively associated with the anogenital distance of male, but not female, newborns [20].

In addition, a statistically significant correlation was found between exposure to DEHP and dibutyl phthalate (DBP) in utero and an increased risk of premature birth [21]. In a study in which 3266 pregnant women participated, in order to investigate the relationship between prenatal exposure to phthalates and the risk of premature birth and gestational age, nonlinear relationships between phthalate metabolites and gestational age were determined. Exposure to some phthalate metabolites has been found to be associated with an increased risk of overall preterm and post-term birth [22].

In 1090 pregnant women in the Puerto Rico Test site for Exploring Contamination Threats (PROTECT) cohort study, the metabolites di-n-butyl phthalate (DBP) and di-isobutyl phthalate (DiBP) were associated with a higher likelihood of preterm birth. The authors explain this association with increased oxidative stress and the inflammatory process due to exposure to certain phthalates and their metabolites. They indicate that this hypothesis is supported by animal and in vitro studies that indicate the ability of some phthalates to cause increased inflammation and oxidative stress [23].

Additionally, because of in utero exposure to phthalates, the so-called “phthalate syndrome” occurs, which includes malformation of the reproductive organs, shortening of the anogenital distance, and nipple retention, and the effects are especially pronounced in male newborns [16]. Research indicates that phthalates because they act pro-inflammatory and increase oxidative stress, can lead to metabolic and cardiac disorders. According to a study conducted on neonatal rat cardiomyocytes, exposure to DEHP leads to metabolic remodeling of cardiomyocytes, whereby heart cells use fatty acids for energy production. Consequently, there is an accumulation of lipid intermediates, lactate, protons, and reactive oxygen species. This connection can lead to an increased sensitivity of the heart to ischemia and the occurrence of ventricular dysfunction [24].

### 3.3. Perfluoroalkyl Chemicals

Perfluoroalkyl chemicals (PFCs) which include perfluoroalkyl acids (PFAAs), including perfluorinated carboxylic acids (PFCAs) and perfluoroalkane sulfonic acids (PFSAs), are used for the purpose of coating and waxing various materials in industry, then as conductors, and very often as an ingredient objects of general use (textiles, paper, leather, fire-fighting foam, and cosmetics) and part of food packaging [25]. Exposure to PFCs during early development is associated with developmental neurotoxicity and disorders of the immune system, hyperuricemia, and attention-deficit/hyperactivity disorder [26]. On the other hand, there are studies that did not establish a link between exposure to PFCs and attention-deficit/hyperactivity disorder [27].

Although the mechanism of action of PFCs is not clearly established, agonistic action on the peroxisome proliferator-activated receptor, alteration of the immune response, and alteration in thyroid hormone signaling are assumed [25,26,27].

However, due to their long persistence in nature (they are considered persistent organic pollutants—POPs), bioaccumulation and potential toxicity, perfluorooctane sulfonic acids (PFOS) and most of their salts are prohibited for use. According to the 2019 Stockholm Convention, in order to reduce POPs, perfluorooctanoic acid (PFOA) and its salts are planned to be phased out [28,29].

Spinach, lettuce, corn, tomatoes, and strawberries easily absorb PFAAs from soil and air, and plant-based foods represent the most significant dietary source of PFAAs. It has been estimated that the exposure of children in China aged 2–5 years to perfluoroalkyl acids is about 60% of the reference dose established by European Food Safety Authority (EFSA) [25]. In the industrial city of Foshan in China, it was determined that children up to the age of seven were significantly more exposed to PFCs, up to 40-fold higher than those reported in China and other countries [26].

Analysis of breast milk samples shows that newborns and infants up to 6 months of age in Spain are not at risk of excessive dietary exposure to PFAAs, as PFAAs were detected in concentrations that are also below the acceptable daily intake established by EFSA [30]. Exposure of children and adults to PFOS and PFOA in Flanders (Belgium) did not exceed tolerable daily intake (TDI) values in a study published in 2012, although the average daily intake of these compounds is three times higher in children than in adults [31]. A study conducted in California shows that the concentration of PFOA is higher in children aged 2–8 years than in their parents. Significant positive predictors for individual serum PFC concentrations in children were the frequency of consumption of fish, hotdogs, chicken croquettes, French fries, chips, and microwave popcorn. It is important to note that the frequency of wearing waterproof clothing was a significant positive predictor and that the concentrations of PFCs in serum in children moderately correlated with the concentration of PFCs in the dust. It can be concluded that, apart from food, food packaging and other environmental sources, such as dust, have a significant contribution to PFC exposure in children [32].

### 3.4. Perchlorate

Perchlorate is naturally present in the environment: it occurs in the atmosphere, it is also found in soil and drinking water, and in this way, it can get into food, primarily of plant origin [33,34]. Additionally, it occurs as a breakdown product of chlorine disinfectants used in the process of purifying drinking water, which is another source of perchlorate in water [34]. As with exposure to pesticides, diet represents the most significant route of exposure to perchlorates [33,35].

Perchlorate disrupts the homeostasis of the hypothalamic-pituitary-thyroid axis by competitive inhibition of iodide uptake and can cause hypothyroidism, and consequently negative effects on the growth and cognitive development of fetuses, infants, and small children [35,36]. For the aforementioned reasons, exposure to perchlorates is recognized as one of the threats to children’s health, and perchlorate is classified as an endocrine disruptor [37].

EFSA proposed a limit of 20 μg kg^−1^ as the limit value of perchlorate content in ready-to-eat food for infants and young children, i.e., 10 μg kg^−1^ for infant formulas [36]. By examining about 100 samples of ready-made, commercial food for infants and children, available on the Italian and Serbian markets, it was determined that in 10% of the samples the perchlorate content was quantified, but in a concentration that does not pose a health risk [34]. It was concluded that the appearance of perchlorate is sporadic and depends on the type of food—it is mostly quantified in ready-made vegetable food [34].

Based on average dietary intake, a study from Canada found that dietary exposure of children aged 1–5 years to perchlorates was below 10 μg/kg BW/day, with higher exposure in younger children [38]. Relative to the reference dose of 0.7 μg/kg/day established by the Environmental Protection Agency, Valentín-Blasini et al. estimated that the perchlorate exposure of breastfed infants was greater than that of infants fed cow’s milk or soy-based formulas [39]. A study that included newborns and infants up to 6 months of age from France points out that the reference dose of 0.7 μg/kg/day was not exceeded in 95% of cases, but that an exceedance may occur if the water used to reconstitute the formula has a higher perchlorate content, and therefore the risk cannot be excluded [40,41]. In China, perchlorate was detected in over 90% of samples of breast milk, infant formula, baby supplementary food, and drinking water; however, according to the exposure assessment based on the tested samples, the authors believe that consumption will not pose a risk to children [35]. According to the results of a study conducted in Kuwait, the population exposure to perchlorate is estimated to be higher than in America, but still less than the reference 0.7 μg/kg body weight (BW)/day. Children aged 3–5 years are the most exposed group, and green leafy vegetables and other vegetables and fruits, respectively, are the foods with the greatest contribution to perchlorate exposure [33]. Green leafy vegetables make a significant contribution to the concentration of perchlorate in urine according to the conclusions of studies conducted in the United States of America (USA) and Austria [37,42].

Based on a representative sample of the USA population, including children aged 6–11 years and adolescents aged 12–19 years, it was concluded that food and drinking water from the tap contribute significantly to the concentration of perchlorate in urine, with food having a greater contribution [42]. For infants and children 3–9 years from Austria, it was estimated that the TDI of 0.3 μg/kg BW/day was not exceeded if the average daily intake was observed; however, if the intake was higher than the average, both groups were at risk of being exposed to perchlorate in a content greater than TDI [37].

### 3.5. Pesticides

Exposure of children to pesticides is a significant problem since it can slow down the development of the nervous system [43,44] and lead to neurobehavioral deficits [43,45,46], impairment of cognitive abilities (lower intelligence quotient (IQ)), attention disorders, altered growth, the occurrence of rarer types of cancer [43,46], respiratory problems (rhinitis, cough, chronic bronchitis, and asthma) and other serious health consequences [46,47].

Children are often exposed to harmful chemical compounds in higher doses than adults: they put their hands in their mouths, spend more time on the floor (and are exposed to pesticide residues from the dust that settles on the floor, and their diet is usually based on a smaller number of foods, predominantly on fruit and milk, which may contain significant amounts of pesticides. Additionally, the caloric intake of children calculated on BMI is up to 2.5 times higher compared to adults, the percentage of absorption is higher, and the degree of metabolism and elimination of harmful compounds is lower, together making the child’s body more vulnerable and susceptible to (harmful) external influences [43,46].

Exposure to pesticides during pregnancy can contribute to improper growth and development of the fetus, and to the birth of an infant with low body weight [46] and abnormal primitive reflexes. Exposure is also associated with the previously mentioned cognitive and motor disorders in children in later childhood [45]. Exposure to organophosphate pesticides is also associated with an increased risk of attention deficit hyperactivity disorder (ADHD) and autism spectrum disorder (ASD) [45,48].

Because of their tendency to interfere with hormone synthesis, kinetics, or function, pesticides are also recognized as EDCs [49]. Organophosphate (OP) pesticides can interfere with the synthesis of insulin-like growth factor 1 (IGF-1) [50]. In a cross-sectional study in the district of Brebes, Indonesia, where agriculture is represented as an activity, it was determined that the prevalence of children with hypothyroidism, with an average age of 9 years, was higher in the group of children in whom OP pesticide metabolites—dialkyl phosphate metabolites (DAP)—were detected in their urine, compared to children in whom the same metabolites were not detected in urine [44].

It is considered that exposure to OP pesticides through food represents the primary source of exposure [51,52] and is a significant source of exposure in the child population as well [53]. A study from France detected residual pesticides in 67% of food samples intended for infants and children up to 3 years of age. Residues were quantified in 27% of baby foods and 60% of common foods. In the upper-bound scenario, toxic reference values were exceeded for dieldrin, lindane, and one propineb metabolite [54].

Cequier et al. in a study from 2016, based on urinary biomarkers, determined that the exposure of mothers and children to OP pesticides from Norway corresponds to the exposure of mothers and children from other countries, where agriculture is a more prevalent activity than in Norway [55]. The authors suggest that the reason for the similar level of exposure to OP pesticides in Norwegian subjects, where it is considered that there is no such exposure at the workplace (agriculture), could be the consumption of imported foods [52].

In a study conducted in Israel, in which children aged 6–11 years participated, it was found that the amount of fruit and vegetable intake correlated with the urinary concentration of DAP metabolites. It was determined that the concentration of DAP metabolites in urine was twice as high in children who consumed cucumbers, compared to children who did not consume them, and that the concentration of DAP metabolites in urine was three times higher in children who consumed apples, compared to children who did not consume. Although the study has certain limitations, the authors suggest that the consumption of fruits and vegetables, especially apples and cucumbers, could be the cause of increased exposure to OP pesticides in children from Israel, whose exposure is higher compared to children from other countries [45].

Holme et al. examined the influence of consumption of fruit, fruit juices, and vegetables on exposure to OP pesticides in children from the USA, from the region of the federal state of Washington, where agriculture is the predominant activity. The respondents were aged 1–7 years and small variations were observed in the consumption of fruits, fruit juices, and vegetables depending on the season and the occupation of the parents (whether the parents are engaged in agriculture or not). A statistically significant positive correlation was found between the frequency of vegetable consumption and the concentration of dimethyl derivatives of OP (DMAP) pesticides in children of farmers, but not in children whose parents are not engaged in agriculture. Such patterns were not observed for the frequency of fruit or vegetable consumption and urinary concentrations of diethyl derivatives of OP pesticides (DEAP) for any of the cohorts, nor for the period of thinning or harvest of crops/plantings of agricultural crops. In the population of children whose parents are not farmers, a positive association was observed between apple consumption and urinary DMAP concentration in the period of crop thinning, while, quite unexpectedly, the opposite trend was observed in the children of farmers, also in the period of crop thinning. The authors conclude that in the examined samples, there is no relationship between data on the subjects’ fruit and vegetable consumption and the concentration of OP metabolites in urine. They believe that in environments where agriculture is an activity, ambient exposure to pesticides can greatly influence the results of testing the relationship between diet and urinary concentrations of metabolites of OP pesticides and that eliminating ambient exposure as a confounding factor in the study is not easily feasible, nor is the estimation of the size of the influence of diet on said exposure [53].

A study from 2001 indicates that in children from North Carolina and Ohio, USA, consumption of fresh apples and fruit juices three times a week or more often is a predictor for increased concentration of the nonspecific metabolite OP pesticide chlorpyrifos; that is, that consumption of chicken and turkey three times a week and more often is a predictor for increased concentrations of the nonspecific metabolite of permethrin, a pyrethroid pesticide (insecticide) [56]. A 2016 study from Spain indicates that consumption of vegetables, legumes, and grains are among the most significant predictors of exposure to pesticides (OPs, pyrethroids, and herbicides) in children aged 5–12 years, but finds no evidence to support potential health risks from exposure pesticides [57]. In a study conducted in New York, USA, the authors showed that the consumption of fruit, cereals, and meat are the most significant predictors of the concentration of metabolites of OP pesticides in the urine of pregnant women: an increased intake of fruit and cereals positively correlates with the concentration of metabolites of OP pesticides, while an increased intake of meat negatively correlates with the concentration metabolites of OP pesticides [58].

Mehta et al. detected p, p’- dichlorodiphenyldichloroethylene (p, p′-DDE) and p, p’-dichlorodiphenyltrichloroethane (p, p′-DDT) in 5% and 4% of breast milk samples from lactating Indian women, respectively. The authors state that the concentrations of the mentioned pesticides are below the maximum allowed residue level and those other authors obtained similar results in studies also conducted in India [59].

Organochlorine pesticides were detected in more than 60% of samples of baby food prepared by Korean mothers, most often p, p′-DDE, p, p′-DDT, β-hexachlorocyclohexane (β-HCH), and p, p′-dichlorodiphenyldichloroethane (p, p′-DDD). The average proportion of p, p′-DDT in the total amount of organochlorine pesticides per sample was twice as high in food samples for 15-month-old children than for 6-month-old infants, most likely due to the use of other foods in the diet of 15-month-old children. Organochlorine pesticides were detected in concentrations below the permissible limit according to the US and Canadian health agencies [60].

The neonicotinoid pesticide imidacloprid, which was introduced as a replacement for organochlorine pesticides, was detected in 15% of fruit, vegetable, and grain samples in a study from India in 2012, and in some samples, it was present in an amount higher than the permitted value, while it was not detected in infant food [61]. In a study from Serbia by Torović et al., the presence of pesticides was detected in about 50% of baby food samples, and the most frequently detected were the neonicotinoid pesticide acetamiprid and the benzimidazole fungicide carbendazim. Baby food samples from domestic producers were, in comparison with baby food samples from foreign producers, in a significantly higher percentage of positive or noncompliant, more often containing a higher number of residues or the maximum allowed number of residues per sample, although based on the risk assessment, the examined samples did not represent the risk of acute and chronic adverse effects of exposure [62].

Similar results were obtained in a study conducted in Taiwan: in samples of cooked food intended for infants and children up to 6 years of age, acetamiprid was the most frequently detected neonicotinoid residual pesticide. Due to higher exposures, acetamiprid and subsequently imidacloprid were recognized as pesticides of interest due to potential adverse health effects. In this study, neonicotinoid pesticides were most often identified in canned fruit, cherry tomatoes, oilseed rape, bael fruit, and baby bok choy. However, the overall conclusion is that the subjects’ exposure to neonicotinoid residual pesticides through food does not pose a health risk, as it is below the limit for acceptable daily intake [63].

As part of the ORGANIKO LIFE+ randomized crossover study, which examined the effect of organic food on the body, children from Cyprus, aged 11–12 years, were divided into two groups. In one group, the intervention, that is, a 40-day diet based on organic food preceded a 40-day period of a conventional diet, while in the other group, the order was reversed. Intake of organic foods for 40 days led to a reduction in exposure to pyrethroid and neonicotinoid pesticides, and to a reduction in biological markers of oxidative stress and inflammation in subjects. This study, therefore, points to the importance of organic food as a relatively simple strategy to reduce children’s exposure to pesticides [64]. The advantages of switching to organic production in the context of the negative consequences of pesticides on health were also recognized by other authors [65].

## 4. Direct Additives in Children’s Nutrition

### 4.1. Nitrates and Nitrites

Nitrates and nitrites are used as preservatives in cured and processed meats, fish, and cheese. Nitrates have harmful effects on the human body only when they are converted into nitrites. Nitrates are inert in mammalian tissues, but symbiotic bacteria that are part of the normal microbiota flora of the oral cavity and gastrointestinal system can reduce them to nitrites [66]. The intake of nitrates and nitrites and their reaction with secondary amines or amides can produce carcinogenic N-nitroso compounds in the body. In 2015, the International Agency for Research on Cancer specifically classified processed meat (which includes meat that has been salted, dried, or otherwise altered to improve flavor and preservation) as a Group 1 human carcinogen. Such meat processing leads to an increased formation of N-nitroso compounds, which are associated with the risk of colon cancer [67].

Increased intake of nitrite-rich processed meat during pregnancy is associated with an increased risk of brain tumors in children. Nitrates can also lead to thyroid dysfunction in the same way as perchlorates. In recent years, the use of alternative sources of preservatives, such as celery powder, in products labeled as “natural” and “organic” has increased. These products may also contain nitrates and nitrites in concentrations that may be equivalent to or greater than those found in traditional products that use sodium-based preservatives [68].

In the context of human health effects, nitrites, nitrates, and related types of nitrogen compounds such as nitric oxide continue to be the subject of increasing scientific controversy. An increase in the content of reactive nitrogen compounds can lead to nitrosative stress—a harmful process that can be an important mediator of damage to cell structures, lipids, cell membranes, proteins, and deoxyribonucleic acid [69].

The consequences of consuming products with increased concentrations of these additives are particularly pronounced in more sensitive population groups, such as the pediatric population. Compared to nitrates, the amount of nitrite that is ingested in the normal diet of adults is relatively small. However, the nitrate–nitrite–NO pathway in infants does not occur in the same way as in adults due to reduced bacterial conversion of nitrate to nitrite in the oral cavity. When it comes to feeding children and infants, nitrites and nitrates can often be found in infant formula and other baby foods. They enter milk formulas through contaminated water used in the industrial preparation of milk formulas. Adequate nutrition in the first year of life is crucial for the development of the nervous, reproductive, digestive, respiratory, and immune systems, and all these functions can be impaired by the presence of nitrates and nitrites in the child’s diet. The most common and most dangerous toxic side effect of these additives in the diet of children aged 0 to 6 months is acquired methemoglobinemia (gray baby syndrome) or worsening of the health condition of children with hereditary methemoglobinemia. Methemoglobin is a form of hemoglobin in which iron is oxidized from divalent to trivalent, which does not have the ability to carry oxygen [70]. Symptoms of methemoglobinemia include headache, pallor, fatigue, weakness, feeling of lack of air, seizures, coma, and death [71].

An increased intake of nitrates and nitrites in the diet is also associated with cancer, hypertension, increased infant mortality, congenital malformations of the central nervous system, diabetes, spontaneous abortions, respiratory tract infections, and immune system disorders [72].

A study conducted in Turkey compared nitrate and nitrite levels in infant formula and biscuits for children aged 0 to 36 months to assess cancer risk. The average exposure to nitrates and nitrites was 0.03 mg/kg BW/day. The concentrations of both additives were higher in formulas compared to biscuits, and it was concluded that exposure to these additives in food represents a potential risk for children’s health, and the highest level of the hazard index is represented in the age group from 0 to 6 months [73]. A study published in 2011 conducted among preschool and school-aged children in Sweden tracked the average intake of nitrite and nitrate in this population. The research was conducted in spring and autumn, in order to exclude possible seasonal differences, in three age groups (preschool children aged 4 years, children aged 8–9 years, and 11–12 years). The concentrations of nitrites and nitrates in meat products, fruits, vegetables, and drinking water were calculated, and in the next step, the daily intake of these additives was calculated in mg/kg BW. The results, taking into account the endogenous conversion of nitrates to nitrites, showed that 12% of children aged 4 years, 3% of children aged 8 to 9 years, and 1% of children aged 11 to 12 years exceeded the total daily intake of nitrites. Compared to other Scandinavian countries, the total intake of nitrite and nitrate among these populations in Sweden was significantly lower, possibly due to the relatively low nitrate content of Swedish vegetables. In addition, the vegetable intake in this study was low compared to the mean vegetable consumption of European children of the same age [74].

An analysis of processed grain-based food and food intended for infants and young children did not determine concentrations of nitrites and nitrates that exceed the maximum values prescribed by the Commission for Food Additives and Flavorings of the European Food Safety Agency [75].

Children’s health is also affected by the mother’s diet during pregnancy and lactation, so nitrates and nitrites can also be found in breast milk [76]. Apart from the negative consequences for the health of the infant, the presence of nitrites in breast milk has a protective role to a certain extent. They can compensate for nitrite deficiency during the early neonatal period until the enterosalivary nitrate–nitrite–nitrogen oxide pathway is established. Breast milk rich in nitrites plays a role in the prevention of neonatal infections and gastrointestinal diseases by ensuring the bioavailability of nitric oxide. Nitric oxide, under a, so far, unexplained mechanism, helps the child adapt to the extrauterine environment [77].

On the other hand, consumption of nitrate-rich drinking water during pregnancy can potentially lead to premature birth [78]. Additionally, the consumption of drinking water rich in nitrates in early childhood is associated with an increased risk of brain tumors in children, adolescents, and young adults [79].

In addition to brain tumors, the development of thyroid cancer is more common. Nitrate is a competitive inhibitor of the Na-I symporter, thus preventing the absorption of iodine in the thyroid gland, which leads to an increase in thyrotropin, which compromises the synthesis of thyroid hormones. Chronic stimulation of the thyroid gland leads to proliferative changes such as hypertrophy, hyperplasia, and neoplasia [80].

Certain studies have shown higher nitrite concentrations in the saliva of male children with autism spectrum disorders. The results showed that nitrite was present in higher concentrations in male children with ASD than in boys with normal neurological development (ND). Additionally, a positive correlation between salivary nitrite and serum nitrate was observed only in children with ADS, but not in the ND group. This preliminary study lays the groundwork for future studies investigating saliva as a noninvasive diagnostic fluid for ASD, as well as the role of NO in autism diagnosis and therapy [81].

Regardless of the fact that most of the research conducted on the concentration of nitrites and nitrates in commercial products for children’s nutrition did not show that the concentrations of these additives exceed the limit values prescribed by law, the previously mentioned research indicates that the excessive consumption of food with the addition of nitrites and nitrites can still represent a risk and to lead to serious consequences for children’s health.

### 4.2. Artificial Food Colorants

Artificial food colorants (AFCs) are added to food and beverages for aesthetic reasons, resulting in brightly colored products that are particularly attractive to children. In some cases, AFCs serve as nutrient substitutes, such as fruit juices that contain little or no fruit. Each synthetic food coloring is marked with an official number that may vary from country to country. The International Numbering System (INS), created by the World Health Organization and the Food and Agriculture Organization, uses the numbers 100 to 199. EFSA also applies INS with the addition of the prefix “E” to denote “Europe”, and this numbering system includes both synthetic food colors (whether they are safe for use or not) and natural food colors. In contrast to the European Union, the numbering systems in the USA prescribed by the Federal Food, Drug, and Cosmetic Act separate synthetically produced colors that have passed a safety check, from colors obtained from natural sources that do not require a safety check for use [82].

The permitted colors are Carmoisine/Azorubine (E 122), Ponceau 4R (E 124), Erythrosine (E 127), Allura Red (E 129), Tartrazine (E 102), Sunset yellow FCF (E110), Indigotine/Indigo Carmine (E132), Brilliant Blue FCF (E 133), and Fast Green FCF (E 143), and the federal law prescribes acceptable daily intakes of these additives expressed in mg/kg BW/day [83]. Studies published recently show that Brilliant Blue (E133) (54.1%) and Tartrazine (E102) (42.3%) are the most used permitted artificial color additives in the various analyzed food items [84].

AFC use is widespread, as demonstrated by a 2016 study conducted in North Carolina. This study assessed the prevalence of AFC-added food products intended for children. It was observed that 41 out of 66 companies marketed products containing AFC, 43.2% of products contained AFC, and the highest percentage of AFC contained candies (96.3%), fruit-flavored snacks (94%), and powdered mixes for drinking (89.7%) [85].

These results are supported by a study conducted in Saudi Arabia on children aged 6–17 years, which shows that the highest percentage of AFC was found in juices and other drinks, as well as in ice cream and cakes [84]. Given that children use products containing AFCs significantly more often than adults, they are also more susceptible to adverse health effects due to the consumption of products containing AFCs, such as hypersensitivity reactions, behavioral and attention disorders in children, and learning difficulties [82,86].

Stevenson et al. found that children with certain polymorphisms in genes for histamine degradation have more serious negative reactions to AFC, given that histamine plays a role as a neurotransmitter, is responsible for alertness, and plays a role in the manifestation of hypersensitivity reactions [87]. The most popular artificial food coloring, tartrazine yellow, was once thought to be a potential trigger for asthma, allergic rhinitis, and urticaria flare-ups in atopic patients. However, a double-blind, placebo-controlled, crossed-over challenge was performed in 26 atopic patients, and there were no appreciable cutaneous, respiratory, or cardiovascular symptoms as compared to placebo [88]. Some AFCs can be genotoxic, while some AFCs are contaminated with benzidine and other carcinogens [89]. Additionally, exposure to artificial food dyes in childhood may contribute to learning disabilities. Prenatal exposure to AFC alters the concentration of N-methyl-D-aspartate receptor subunits involved in learning and memory formation processes [90].

### 4.3. Monosodium Glutamate—A Flavor Enhancer

Aroma enhancers are additives that stimulate the action of already present food aromas and thereby enable the achievement of more pronounced aromatic properties of food [91].

Monosodium glutamate is the sodium salt of glutamic acid and is an integral part of some plant and animal proteins and makes up 10–25% of all proteins in human food [91]. It is mostly found naturally in foods, such as tomatoes and cheese. As an additive, it is added to soup cubes, soy sauces, stews, spices, salty snacks, etc. [92]. Monosodium glutamate is used primarily as an umami (meaty and salty) flavor enhancer [93,94]. One of the most controversial food additives that the Food and Drug Administration has allowed for use is monosodium glutamate. Although it is “generally regarded as safe” (GRAS) by regulatory agencies to be used in the food supply, some research indicates that it may have a negative health impact [92,95,96,97,98,99,100,101].

Glutamic acid is easily metabolized in the human body [92]. Since glutamate is an important neurotransmitter, some studies show that its excess intake may pose a health risk, especially to children. Neurotoxicity caused by excessive activation of glutamate receptors is associated with the risk of developing acute and chronic neurodegenerative diseases such as amyotrophic lateral sclerosis, Alzheimer’s disease, drug addiction, and schizophrenia [95].

Glutamic acid has two COOH groups that, under certain pH conditions, can be found as zwitter ions and bind two sodium cations. The goal of using glutamate is to reduce sodium chloride intake by enhancing the salty taste [91]. However, excessive consumption of glutamate can lead to excessive intake of sodium (salt poisoning) and trigger the so-called Chinese restaurant syndrome, characterized by symptoms that include sweating, headache, flushing, and in more severe cases, throat swelling and chest pain [96].

Excessive sodium intake may pose a risk for hypertension in children and adults, and of particular concern is the increased sodium intake among the population older than two years [97,98,99].

Some studies also point out that glutamate can cause bronchospasm and lead to asthma exacerbation or migraine [100,101].

### 4.4. Aspartame—Sweetener

Food additives that are used to improve sweetness with or without adding extra calories are called sweeteners [102,103,104,105]. Aspartame (E-951) is an artificial sweetener used as a substitute for sugar and it is present in several sugar-free products, as well as in some medications and vitamin supplements [102,103,104].

It is added to nonalcoholic and alcoholic beverages, which most often bear the indication “zero sugar” [105].

In the intestine, aspartame undergoes complete breakdown into phenylalanine, aspartic acid, and methanol, which are further absorbed, metabolized, and excreted. Aspartame is contraindicated for use in people who have reduced activity of the phenylalanine hydroxylase enzyme that needs to metabolize phenylalanine. The conversion of phenylalanine to tyrosine is successful, so phenylalanine accumulates in the blood and brain, which can lead to negative consequences for the child’s neurological development. If a deficiency of this enzyme is determined, absolute restriction of the consumption of all products containing phenylalanine or compounds that are metabolized to phenylalanine in the body is advised [105]. The safety of aspartame has been studied since its discovery and is one of the most rigorously tested food ingredients [106]. Despite its extensive use, there are still questions about its safety [107,108].

There are scientific studies that point to the potential association of aspartame with serious health problems, including the occurrence of tumors, cardiovascular disease, epilepsy, stroke, dementia, and Alzheimer’s disease [106,109]. The association of aspartame intake with increased frequency of mood disorders and headaches was also observed, but this was not confirmed among children [110,111]. A recent study found a correlation between consumption of aspartame in artificially sweetened soft drinks and early menarche [112].

One case study conducted on a child (11 years old), proposed a connection between systemic contact dermatitis and consummation of montelukast chewable tablets (which contained aspartame) and usage of all personal health products containing aspartame [113].

The previously presented data justify the question of the risk of adding aspartame to “diet” drinks or slimming products. On the other hand, over 100 regulatory agencies in developed countries, including the US Food and Drug Administration, the British Food Standards Agency, and the European Food Safety Agency, consider aspartame safe for human use in recommended amounts of consumption [106,109]. Since the currently available data are ambiguous when it comes to the safety of aspartame consumption, additional follow-up studies are necessary in order to evaluate potential benefits and risks.

## 5. Limitations

Since we performed a narrative review, there is a reasonable possibility that not all available evidence on the topic was considered, even though three remarkable health-related electronic databases were included in the search. Our main objective was to discuss some of the most concerning additives children have high exposure to. Thus, we did not discuss all classes of additives. It can be said that the limitation is that the review refers only to the child population. We believe that a systematic review on the subject, including an adult population, would be a significant contribution to scientific and other relevant communities.

## 6. Conclusions

The use of food additives is justified only when it serves a well-defined technological function, when it is limited, and does not adversely affect the nutritional value of food, without posing a health risk to consumers. Of the numerous additives in use, bisphenols, phthalates, perfluoroalkyl chemicals, perchlorates, pesticides, nitrates and nitrites, artificial food colors, monosodium glutamate, and aspartame are most often found in food consumed by children. Due to the still-insufficient development of the children’s bodies, they are more susceptible to the harmful effects of additives than adults. That is why it is important to carry out continuous education of parents and young people with the aim of increasing literacy about the presence and potential risk of additives present in food for children. Competent authorities have a central function in the reduction in risks and the prevention of negative health consequences; therefore, it is necessary to actively monitor the appearance of newly registered additives and harmonize existing legal regulations. It is necessary to continuously conduct research in order to more clearly determine the impact of additives on children’s health, considering their wide distribution and daily use.

## Figures and Tables

**Table 1:** Summary of food-related uses, the proposed mechanism of action, and health concerns for the additives discussed in this review.

	Additive	Use	Proposed Mechanism of Action	Intake Risk
Indirect	Bisphenols	Coating of primary packaging, cans, and bottles	Endocrine disruptors (interfere with the synthesis, secretion, transport, metabolism, site of action, or elimination of hormones)	Polycystic ovary syndrome, obesity, and increase in cardiometabolic risk factors
	Phthalates	Lubricants, adhesives, and plasticizers used during the packaging manufacturing process	Metabolism of phthalates produces products that affect the expression of peroxisome proliferator-activated receptors (PPARs), which play a significant role in the metabolism of lipids and carbohydrates	Insulin resistance, disorders in the development of the male genital system, premature birth, metabolic disorders, and cardiac disorders
	Perfluoroalkyl chemicals	They are used for coating and waxing various materials in the industry, as an ingredient in items of general use (textiles, paper, leather, fire extinguishing foam, and cosmetics), as part of food packaging, and persistent organic pollutants that bioaccumulate in fruits and vegetables	An agonistic effect on the peroxisome proliferator-activated receptor, alteration of the immune response, and alteration in thyroid hormone signaling	Developmental neurotoxicity, immune system disorder, hyperuricemia, and attention-deficit/hyperactivity disorder
	Perchlorate	Naturally present in the environment (atmosphere, soil, and drinking water) thus gets into food of plant origin, and is a breakdown product of chlorine disinfectants used in the process of purifying drinking water	It disrupts the homeostasis of the hypothalamic–pituitary–thyroid axis by competitive inhibition of iodide uptake (endocrine disruptor)	Hypothyroidism; negative effects on growth and cognitive development of fetuses, infants, and young children
	Pesticides	Exposure to pesticide residues from dust that settles on the floor; a diet based on fruit, milk, and grains that may contain significant amounts of pesticides	Interfering with the synthesis, kinetics, or function of hormones (endocrine disruptors); organophosphate pesticides can interfere with the synthesis of insulin-like growth factor 1	It can slow down the development of the nervous system and lead to neurobehavioral deficits, impaired cognitive abilities (lower IQ), attention deficit disorder, altered growth, the appearance of some types of carcinomas, and respiratory problems (rhinitis, cough, chronic bronchitis, and asthma)
Direct	Nitrates and nitrites	Preservatives in dried and processed meat, fish and cheese, milk formula, biscuits and other snacks; contaminated water	By reaction with secondary amines or amides, carcinogenic N-nitroso compounds can be formed, nitrosated stress damage to cell structures, lipids, cell membrane, proteins, Na-I symporter disruptor	Increased risk of colon cancer, brain tumors in children, thyroid gland dysfunction, methemoglobinemia, premature birth of a child, and disorders of the autistic spectrum
	Artificial food colorants (AFC)	Added to food and drinks for aesthetic reasons	Individuals with polymorphisms in genes for histamine degradation have more severe adverse reactions to AFC	Hypersensitivity reactions, behavioral and attention disorders in children
	Monosodium glutamate	It can be found naturally in tomatoes and cheese, and as an additive, it is added to soup cubes, soy sauces, stews, spices, salty snacks…	Sodium overdose due to excessive consumption, and neurotoxicity caused by excessive activation of glutamate receptors	Increased risk of acute and chronic neurodegenerative diseases (amyotrophic lateral sclerosis, Alzheimer’s disease, drug addiction, and schizophrenia), hypertension, bronchospasm, and Chinese restaurant syndrome
	Aspartame	Artificial sweeteners present in several sugar-free products (beverages), as well as in some medications and vitamin supplements	Deficiency of the phenylalanine hydroxylase enzyme leads to the accumulation of phenylalanine and the manifestation of toxic effects	Negative consequences on the child’s neurological development due to the toxic effect of phenylalanine, increased frequency of mood disorders, and frequent headaches

## Data Availability

Not applicable.
